# High abundance of the colistin resistance gene *mcr-1* in chicken gut-bacteria in Bangladesh

**DOI:** 10.1038/s41598-020-74402-4

**Published:** 2020-10-14

**Authors:** Salequl Islam, Umme Laila Urmi, Masud Rana, Fahmida Sultana, Nusrat Jahan, Billal Hossain, Samiul Iqbal, Md. Moyazzem Hossain, Abu Syed Md. Mosaddek, Shamsun Nahar

**Affiliations:** 1 Department of Microbiology, Jahangirnagar University, Savar, Dhaka, 1342 Bangladesh; 2 Department of Oral Maxillofacial Surgery, Faculty of Dentistry, BSMMU, Dhaka, 1210 Bangladesh; 3 Department of Statistics, Jahangirnagar University, Savar, Dhaka, 1342 Bangladesh; 4 Department of Pharmacology, Uttara Adhunik Medical College, Uttara, Dhaka, 1230 Bangladesh

**Keywords:** Antimicrobial resistance, Epidemiology

## Abstract

Colistin is considered a last-resort reserved drug for the treatment of critical human infections by Gram-negative bacteria. Phenotypic colistin-resistance is strongly associated with plasmid-mediated mobile colistin resistance (*mcr*) genes. The *mcr*-bearing Enterobacteriaceae have been detected in many countries from environments, animals, and humans. This study investigated phenotypic colistin-resistance and the distribution of *mcr-1, mcr-2, mcr-3, mcr-4, and mcr-5* genes in chicken-gut bacteria in Bangladesh. Bacteria were isolated from poultry- and native-chicken droppings, and their susceptibilities to colistin were determined by agar dilution and *E-test* minimal inhibitory concentration (MIC) measurements. Multiplex polymerase chain reactions detected *mcr-1 to mcr-5* genes. Overall, 61.7% (92/149) of the isolates showed colistin resistance by agar dilution assessment (MIC > 2.0 μg/mL). The phenotypic resistance was observed considerably higher in poultry-chicken isolates (64.6%, 64/99) than in native-chicken isolates (56%, 28/50; *p* = 0.373). All the resistant isolates showed MIC levels between > 2 and > 128 μg/mL. The *mcr*-genes (*mcr-1*and *mcr-2* combined*)* were detected more in poultry gut bacteria (36.4%) than native-chicken isolates (20%, *p* = 0.06). Despite bacteria sources, *mcr-*genes appeared to be significantly associated with phenotypic colistin-resistance phenomena (*p* < 0.001). Prior colistin usage led to a substantial increase in the proportion of bacteria with *mcr-*genes and phenotypic resistance (*p* < 0.001).

## Introduction

Emerging antimicrobial resistance (AMR) is a leading public health concern worldwide^1^. Multiple studies have reported a strong association of antimicrobial usage (AMU) in animals with the extensive AMR burden^2,3^. Colistin (polymyxin E) has been used worldwide in food animals for decades for therapeutic, prophylactic, and growth promotion purposes^4–6^. China and Brazil were two major users of colistin as a growth promoter in agriculture and livestock^7,8^, until a recent ban due to AMR as a health hazards^9^. However, the ban in China includes the prohibition of using colistin as a feed additive only, and its use as a therapeutic agent for sick animals is not prohibited^10^. In most Asian countries, including India, Japan, Korea, and Vietnam, colistin has been used extensively as an feed-administered animal growth promoter to increase the body weights of farm animals^11,12^. The indiscriminate presence of therapeutic or prophylactic antimicrobials in either humans or animal husbandry generates selection pressure and influences the plausible development of AMR in pathogens and other diverse commensal microbial populations^13,14^. The presence of residual antimicrobials in farmed animal foods has been evidenced by multiple studies^15,16^, and is a major human health concern. Acquisition of the plasmid-mediated mobilized colistin resistance gene variant-1 (*mcr-1*) was first described in Enterobacteriaceae from both farm-animal products and humans^7^. After that, multiple *mcr-1*-carrying species of Enterobacteriaceae were detected in many countries in the environment, animals, and humans^17–19^. Subsequently, more variants of transferable colistin resistance *mcr* gene (*mcr-1* to *mcr-9*) have been described in Enterobacteriaceae^20,21^. As colistin was extensively used in animal production in China, increased colistin resistance has been observed there in recent years^18,22^. The outcomes further attest to the relationship between colistin usage and the acquisition of antimicrobial-resistant bacteria from food-producing animals^13,14^. Moreover, colistin heteroresistance (wherein traditionally susceptible pathogens eventually appear resistant) has also been reported for many pathogens^23–25^. The *mcr-1* gene was found in human pathogens with and without a history of colistin treatment^26–28^. A recent outbreak with colistin-resistant pathogens ended a very high case-fatality rate in humans^29^. Thus, identification of the root cause, transmission, and trajectories of colistin-resistant infection has drawn worldwide attention. Colistin has been approved as a veterinary medicine and as a feed additive in Bangladesh. To our knowledge, no known study has reported either the prevalence of colistin usage in Bangladeshi food animals or the association of colistin-usage in food animals with evolution of corresponding drug-resistant pathogens. This study investigated the prevalence of the *mcr-1* to *mcr-5* genes linked to phenotypic colistin resistance in farm-origin poultry chicken bacteria and household-dwelling native chicken isolates in Bangladesh.

## Results

### Study farms and samples

**Figure 1:**
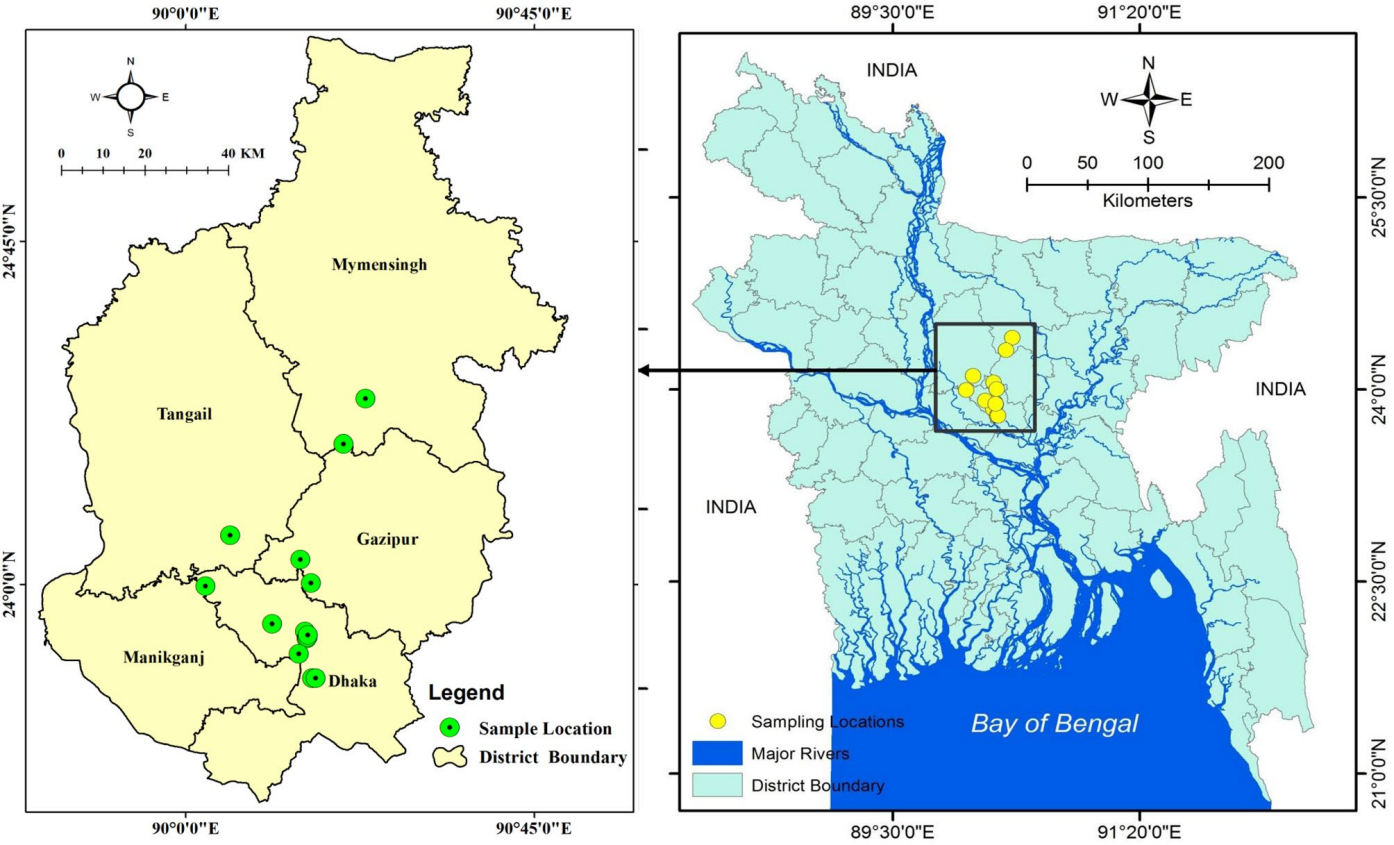
Sampling areas. Spatial locations of poultry farms and houses were shown from where samples of poultry-chicken droppings (n = 100) and native-chicken droppings (n = 50) were collected. Sampling locations covering five districts in Bangladesh are indicated in the map. We used geographic information mapping software, ArcGIS version 10 for Windows to draw the sampling spot-location map.

A total of 105 poultry dropping samples from 21 farms were collected for this study. After routine cross-checking, five samples from one farm were discarded due to incompleteness of the exposure history data. Thus, 100 faeces from 20 poultry farms located in five districts of Bangladesh were screened for distinct types of enteric bacteria (Fig. 1). The selected farms were a combination of small (capacity 50–2000 chickens), medium (2001–5000 chickens), and large (5000+ chickens) farms. More than half of the farmed chickens were intended for meat production, namely, broiler chickens (6/20, 30%) and Pakistani cocks (30%). The remaining 40% (8/20) of the chickens were layer type, and were farmed primarily for egg production. Layer chickens are also used as meat sources when egg production rates are low. Colistin usage was reported in 66.7% of the meat-producing chickens (8/12) and 37.5% of the layer chickens (3/8) in the PFs. Over 80% of the farm managers in this study had no professional training regarding the dosing of different veterinary medicines and their potential side-effects. However, the managers were familiar with different brand names of ‘over the counter’ (OTC) antimicrobials and growth-promoting drugs used for chickens. Our data collector captured the brand names as original data and subsequently translated to generic names before entry into the database. Other 25 native-chicken faeces were collected from five individual houses and investigated in the study. None of the farmers who provided droppings of native chickens had profession training for animal husbandry farming.

### Isolation and identification of chicken faecal bacteria

The poultry faeces yielded 375 different isolates, and native faeces generated 50 isolates (Supplementary Fig. 1A). The poultry faeces carried more varieties of bacteria than the native chicken faeces. Of the total isolates, 99 were randomly selected from poultry bacteria, and all the native bacteria were subjected to assessment of colistin susceptibility and *mcr-1* to *mcr-5* carriage. Overall, all the poultry- and native-chicken faeces yielded at least one type of bacteria. Very few culture plates exhibited no growth. The 99 isolates from poultry faeces were classified as, 21 *E. coli* (21.2%), 36 *Proteus* spp. (36.4%), 20 *Klebsiella* spp. (20.2%), 10 *Salmonella* spp. (10.1%), 3 *Shigella* spp. (3.0%) and 9 *Enterobacter* spp. (9.1%). The native chicken faeces yielded 13 *E. coli* (26.0%), 16 *Proteus* spp. (32.0%), 10 *Klebsiella* spp. (20.0%), 6 *Salmonella* spp. (12.0%), and 5 *Enterobacter* spp. (10.0%), but no *Shigella* spp.

### Phenotypic colistin susceptibility assessment

**Figure 2:**
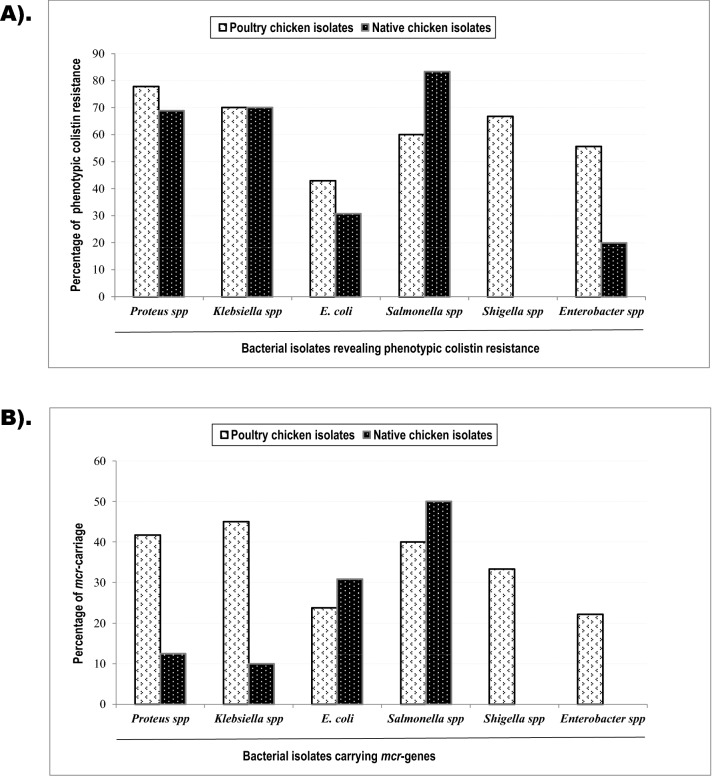
Isolate-wise phenotypic colistin resistance and *mcr*-gene carriage. (**A**) The agar dilution methods evaluated phenotypic colistin resistance. The bacteria were considered resistant when their minimum inhibitory concentrations (MICs) appeared > 2 μg/mL. Bacterial species examined for the phenotypic resistance were presented on the X-axis. The white-coloured bars in the Y-axis indicate the percentage of colistin resistance exhibited by poultry isolates. Likely, the black-coloured bars illustrate the percentages of resistant isolates from native chicken faeces. Percentile distributions of the poultry isolates show similar levels of colistin phenotypic resistance compared to native chicken isolates. (**B**) Carriage of the *mcr* genes (*mcr-1* to *mcr-5*) was examined in chicken gut bacteria by polymerase chain reaction (PCR). The percentage of the identified *mcr*-genes in poultry-chicken bacteria was compared to that of native chicken bacteria. White bars to the Y-axis represent the proportions of the *mcr*-genes in poultry isolates, and black bars represent the native-chicken bacteria. Percentile distributions show the poultry-chicken isolates carried more *mcr* genes than that of the native chicken isolates.

Following EUCAST guidelines, isolates were considered susceptible (S) when the MIC values exhibited ≤ 2 μg/mL and resistant (R) when MICs appeared > 2 μg/mL. (Supplementary Fig. 1B). Experiments were repeated when some single colonies or a thin haze growth was observed within the inoculated spot. Concordant results were found in independent MIC assessment by *E-test* (Supplementary Fig. 1C). A total of 92 (61.7%) isolates showed colistin-resistance by the agar dilution test. Of these, 64 (64.6%) were poultry chicken isolates compared to 28 (56%) from the native group (Table 1, *p* = 0.373). Isolates from poultry- and native-chicken faeces exhibited overall indifferent phenotypic colistin resistance (Fig. 2A). Intraspecies bacteria from the two sources demonstrated similar phenotypic resistance levels (Supplementary Table 1).

**Table 1:** Phenotypic colistin susceptibilities and *mcr*-carriage in poultry- and native-chicken isolates.

Source (isolate number)	Phenotypic colistin susceptibility, number (%)			Carriage of *mcr* gene, number (%)		
	Sensitive	Resistant	*p* value	Yes	No	*p* value
Poultry chicken (n = 99)	35 (35.4)	64 (64.6)	*0.373*	36 (36.4)	63 (63.6)	*0.060*
Native chicken (n = 50)	22 (44)	28 (56)		10 (20.0)	40 (80.0)	
Total (N = 149)	57 (38.3)	92 (61.7)		46 (30.9)	103 (69.1)	

Italics are used for ‘bacterial names’ as a standard rule in scientific nomenclature.

### Detection of the *mcr* gene variants

The colistin resistance genes, *mcr-1* to *mcr-5* were assessed in 149 chicken isolates using PCR analysis. The *mcr-1* and *mcr-2* genes were detected in 43 (28.9%) and 5 (3.4%) of isolates, respectively, from a combination of poultry- and native-chicken faeces (Supplementary Fig. 1D). Of them, two isolates carried *mcr-1* and *mcr-2* concurrently, and the remaining were mutually exclusive. None of the isolates was detected with *mcr-3, mcr-4,* and *mcr-5*. Separately, *mcr-1* was found in 36.4% of the poultry bacteria (36/99) and 20% of the native chicken isolates (10/50). A statistically significant and moderately strong correlation was observed between *mcr-genes* and poultry faeces-origin bacteria (r = 0.167, *p* = 0.041). In contrast, no marked differences were observed in the carriage of *mcr-2*: only two isolates (2%) from poultry-chicken faeces and three (6%) from native-chicken faeces carried the allele (*p* = 0.335). The *mcr*-bearing isolates were detected in all five districts located in two divisions, namely, Savar (n = 20), Manikganj (n = 9), Tangail (n = 6), Mymensingh (n = 4), and Gazipur (n = 2) (Fig. 1). In this study, 17 *Proteus* spp. 10 *Klebsiella* spp. nine *E. coli*, seven *Salmonella* spp. one *Shigella* spp. and two *Enterobacter* isolate harboured the *mcr-1* gene. The *mcr* trait was found in overall higher proportion in poultry-chicken isolates than in native chicken isolates (Fig. 2B). However, Chi-square analysis showed a statistically non-significance difference in the overall *mcr*-carriages in isolates from the two different origins (*p* = 0.06). The separate intraspecies presence of *mcr* was not significantly different between the two groups. (Supplementary Table 1).

Sequence analyses of the amplified *mcr-1* gene showed high identity with global *mcr-1* gene sequences in the NCBI database. A majority of the nucleotide sequences among the three clones were identical, except for a four-nucleotide mismatch with one clone (Supplementary Fig. 2A). The four-nucleotide substitutions appeared to be non-synonymous, which changed four amino acid substitutions in one bacterial *mcr-1* (Supplementary Fig. 2B).

### Association of the *mcr* and phenotypic colistin resistance

**Figure 3:**
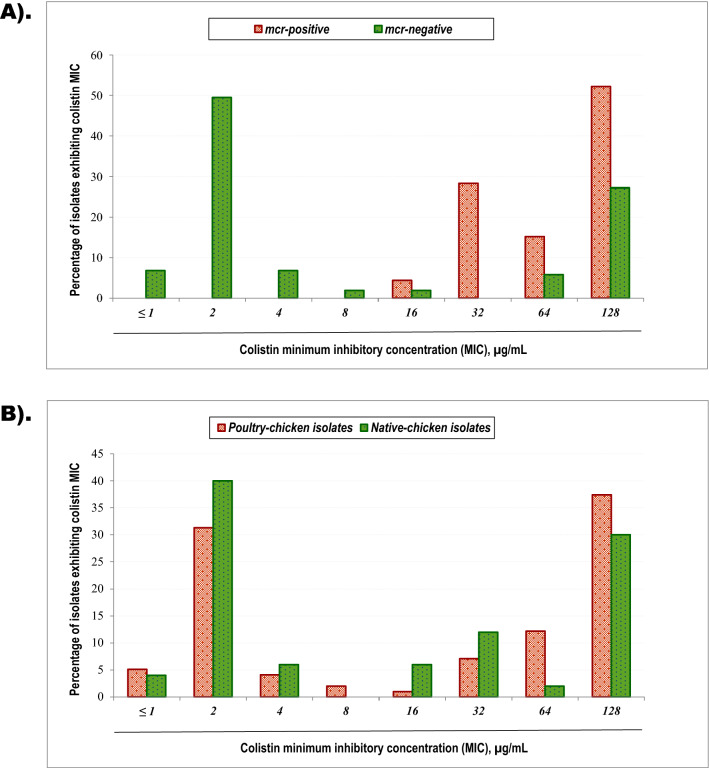
Minimum inhibitory concentrations (MICs) of isolates based on the *mcr*-gene acquisition and sources of origin. All the bacteria in this study were subjected to measure minimum inhibitory concentrations (MICs) of colistin between 1.0 and 128.0 μg/mL by agar dilution tests. Percentages of bacteria exhibiting each MIC level were calculated. The X-axis shows each of the MIC points, and Y-axis represents the percentages of isolates at a particular MIC. (**A**) The percentages of *mcr*-positive and *mcr*-negative isolates at MIC endpoints were compared. Red-coloured bars show the magnitudes of *mcr*-positive isolates, and the green-coloured bars are for *mcr*-negative. Percentile distributions show that *mcr*-positive isolates exhibited higher MICs compared to *mcr*-negative isolates. (**B**) The proportions of poultry- and native-chicken isolates at particular MIC points were compared. Red-coloured bars show the percentages of poultry-chicken origin isolates, and the green-coloured bars are for native-chicken bacteria. No marked differences of MIC were observed in isolates categorized in two source groups.

A strongly significant association was observed between *mcr-1* and colistin-resistant phenotypes by the agar dilution test (*p* < 0.001). All the five isolates carrying *mcr-2* were phenotypically colistin-resistance; however, no statistically significant association between *mcr-2* and phenotypically colistin-resistance was observed (*p* = 0.064). The lost statistical significance could be attributable to the small sample sizes. By combining the two variants' acquisition, isolates possessing the *mcr *demonstrated a very strong harmony to the phenotypic resistance attribute (*p* < 0.001). All of the isolates carrying *mcr* trait showed colistin MIC levels between16 and > 128 μg/mL (Fig. 3A). Unexpectedly, some *mcr*-negative isolates (44.7%) also showed phenotypic colistin-resistance by the agar dilution test (Supplementary Table 2). The finding indicates the presence of some other factors besides the identified *mcr* variants to contribute colistin resistance to the isolates.

**Table 2:** Association of prior colistin exposure with phenotypic colistin susceptibility and *mcr* carriage in poultry- and native-chicken isolates.

Origin of isolates	Prior colistin exposure (N)	Phenotypic colistin susceptibility, number (%)			Carriage of *mcr* gene, number (%)		
		Sensitive	Resistant	*p* value	Yes	No	*p* value
Poultry-chicken faeces	Yes (61)	14 (23.0)	47 (77.0)	0.002	28 (45.9)	33 (54.1)	0.018
	No (38)	21 (55.3)	17 (44.7)		8 (21.1)	30 (78.9)	
Native-chicken faeces	Yes (10)	2 (20.0)	8 (80.0)	0.154	5 (50.0)	5 (50.0)	0.018
	No (40)	20 (50.0)	20 (50.0)		5 (12.5)	35 (87.5)	
Combined	Yes (71)	16 (22.5)	55 (77.5)	0.000	33 (46.5)	38(53.5)	0.000
	No (78)	41 (52.6)	37 (47.4)		13 (16.7)	65 (83.3)	

### Association of prior colistin usage with its phenotypic resistance and *mcr* acquisition

Twelve poultry farms (60%, 12/20) reported using colistin sulphate for the purposes of treatment or growth promotion. One lot of the household chickens had a history of colistin usage (20%, 1/50). The 12 farms with colistin exposure yielded 71 isolates, of which 33 were *mcr*-positive (46.5%). In contrast, the droppings from the remaining non-exposed chickens yielded 16.7% *mcr* harbouring isolates (13/78). Therefore, a substantially higher proportion of *mcr* was observed in colistin-exposed bacteria (*p* < 0.001). Similar higher proportion of phenotypic colistin resistance was noted in the colistin-exposed group of isolates (*p* < 0.001). Statistical significance became attenuated for phenotypic resistance and *mcr*-acquisition in separate analyses for poultry- and native-chicken isolates separately (Table 2). It is likely that the reduced sample sizes in the separate analyses led to loss of statistical significance.

### Minimum inhibitory concentration determination

Entire isolates were subjected to MIC measurements by agar dilution tests, in which > 2 μg/mL is considered a clinical breakpoint for colistin resistance. An overall consistent result was observed between the agar dilution and the *E-test* methods for colistin MIC determination. Each time, the control plate without colistin sulfate showed adequate growth of both test-isolate and susceptible control *Escherichia coli* ATCC25922 strains. For several isolates, mismatching of two- to four-fold MIC values were observed between *Etest* and dilution methods. Consistent MIC patterns were observed in *mcr-1/mcr-2 *carrying isolates: the bacteria's growth was found from the lowest concentration of 1.0 μg/ml colistin to their MICs. All the *mcr*-bearing isolates showed higher MIC ranging between > 16 and > 128 μg/mL (Fig. 3A). However, some inconsistent ‘skipped’ (no growth) patterns were observed for several *mcr*-naive isolates: no growths were observed at lower colistin concentrations, such as 1–4 μg/mL, but growth appeared at immediate higher concentration (8 μg/mL). We repeated the experiments and considered the MIC values based on the growth observed at the highest concentrations. Although most *mcr*-naïve isolates demonstrated lower colistin MICs, a portion had shown seriously high MIC levels (Fig. 3A). With the acquisition of *mcr*-gene, each type of bacteria, such as *Proteus* spp., *Klebsiella* spp., *E. coli, Salmonella* spp., and *Enterobacter* spp., revealed higher MIC endpoints (Table 3). On the other hand, isolates from poultry-chicken faeces exhibited an almost indifferent MIC levels with the isolates from native-chicken droppings (Fig. 3B). Further, independent intraspecies MIC manifestations from the two sources appeared reasonably similar (Table 4).

**Table 3:** Distribution of colistin minimum inhibitory concentrations (MICs) of *mcr*-positive and *mcr*-naïve isolates.

Organism tested	*mcr* presence	No. of isolates	Colistin MIC (μg/mL), n (%)								*p* value
			≤ 1.0	2.0	4.0	8.0	16.0	32.0	64.0	128.0	
*Proteus* spp.	*mcr*-positive	17	–	–	–	–	1 (5.9)	1 (5.9)	4 (23.5)	11 (64.7)	*0.068*
	*mcr*-negative	35	2 (5.7)	11 (31.4)	–	–	2 (5.7)	–	4 (11.4)	16 (45.7)	
*Klebsiella* spp.	*mcr*-positive	10	–	–	–	–	–	3 (30.0)	1 (10.0)	6 (60.0)	*0.012*
	*mcr*-negative	20	–	9 (45.0)	–	–	–	–	1 (5.0)	10 (50.0)	
*E. coli*	*mcr*-positive	9	–	–	–	–	–	6 (66.7)	–	3 (33.3)	*0.000*
	*mcr*-negative	25	4 (16.0)	17 (68.0)	2 (8.0)	1 (4.0)	–	–	1 (4.0)	–	
*Salmonella* spp.	*mcr*-positive	7	–	–	–	–	–	3 (42.9)	1 (14.3)	3 (42.9)	*0.024*
	*mcr*-negative	9	1 (11.1)	4 (44.4)	3 (33.3)	–	–	–	–	1 (11.1)	
*Shigella* spp.	*mcr*-positive	1	–	–	–	–	–	–	–	1 (100)	*0.223*
	*mcr*-negative	2	–	1 (50.0)	–	1 (50.0)	–	–	–	–	
*Enterobacter* spp.	*mcr*-positive	2	–	–	–	–	1 (50.0)	–	1 (50.0)	–	*0.007*
	*mcr*-negative	12	–	9 (75.0)	2 (16.7)	–	–	–	–	1 (8.3)	

Italics are used for ‘bacterial names’ as a standard rule in scientific nomenclature.

**Table 4:** Distribution of colistin minimum inhibitory concentrations (MICs) of poultry- and native-chicken isolates.

Organism tested	Origin of isolates	No. of isolates	Colistin MIC (μg/mL), n (%)								*p* value
			≤ 1.0	2.0	4.0	8.0	16.0	32.0	64.0	128.0	
*Proteus* spp.	Poultry chicken	36	2 (5.6)	6 (16.7)	–	–	–	1 (2.8)	7 (19.4)	20 (55.6)	0.057
	Native chicken	16	–	5 (31.6)	–	–	3 (18.8)	–	1 (6.3)	7 (43.8)	
*Klebsiella* spp.	Poultry chicken	20	–	6 (30)	–	–	–	3 (15)	2 (10)	9 (45)	0.350
	Native chicken	10	–	3 (30)	–	–	–	–	–	7 (70)	
*E. coli*	Poultry chicken	21	2 (9.5)	10 (47.6)	2 (9.5)	1 (4.8)	–	2 (9.5)	1 (4.8)	3 (14.3)	0.351
	Native chicken	13	2 (15.4)	7 (53.8)	–	–	–	4 (30.8)	–	–	
*Salmonella* spp*.*	Poultry chicken	10	1 (10)	3 (30)	1 (10)	–	–	1 (10.0)	1 (10)	3 (30)	0.562
	Native chicken	6		1 (16.7)	2 (33.3)	–	–	2 (33.3)	–	1 (16.7)	
*Shigella* spp.	Poultry chicken	3	–	1 (33.3)	–	1 (33.3)	–	–	–	1 (33.3)	–
	Native chicken	0									
*Enterobacter* spp.	Poultry chicken	9	–	5 (55.6)	1 (11.1)	–	1 (11.1)	–	1 (11.1)	1 (11.1)	0.790
	Native chicken	5	–	4 (80)	1 (20)	–	–	–	–	–	

## Discussion

We investigated the gut flora of both farm-based poultry chickens and home-based cage-free chickens for the prevalence of the five *mcr* gene variants, *mcr-1* to *mcr-5,* which is linked to phenotypic colistin resistance in Bangladesh. The study showed that approximately one-third of the isolates from poultry chicken droppings carried the hazardous *mcr-1* gene. Carriage of *mcr-2* variants was found much lower (3–4%) in bacteria from both poultry- and native-chicken droppings. Some co-occurrences of *mcr-1* and *mcr-2* were also observed. None of the isolates were detected with the presence of *mcr-3, mcr-4*, and *mcr-5*. The findings were consistent with earlier reports of the *mcr* gene in some GNB isolated from food animals in several countries^7,30^. Furthermore, we detected the *mcr-*gene in native chicken gut isolates as well, although at marginally lower proportions. The *mcr-*habouring bacteria, regardless of specimen source, were also found to be resistant to colistin by the agar dilution method. Moreover, the determined MIC values for all *mcr-*positive isolates were between > 16 and ≥ 128 μg/mL, which confirmed the association of *mcr* genes with colistin resistance. To the best of our knowledge, there is no scientific information available about colistin-resistant bacteria identified from either livestock or the clinical sector in Bangladesh. One brief report described *mcr-1* from sewage samples in Bangladesh^31^. Our study is the first of its kind, describing carriage of the colistin-resistance *mcr* gene in both farmed poultry and cage-free native chicken bacteria in Bangladeshi settings. As anticipated, occurrences of the *mcr* genes, singly or a co-existence of the two (*mcr-1* and *mcr-2*) were associated with an increased phenotypic colistin resistance (higher MIC values). A combination of the two genes may enable bacteria to accumulate multiple genes simultaneously to emerge as high-risk enteric bacterial clones easily. Multiple studies have reported that the co-occurrences of multiple clinically relevant antibiotic-resistant genes with *mcr *into the same bacteria possess increased resistance to different antimicrobials^32,33^. On the other hand, a significant portion of isolates without carrying any detectable *mcr* gene, showed phenotypic resistance to colistin. The inconsistency of the genotype–phenotype association could be explained by other variants of *mcr* genes or factors that have not been investigated in this study^21^. Mutational inactivation in the essential chromosomal gene, *mgrB* could further explain the colistin-resistant isolates without the carriage of *mcr* genes^34^. More studies will be required to find some mechanistic explanations for the colistin phenotypic discrepancy to *mcr* genes. This study identified *mcr-*bearing colistin resistant enteric bacteria in five different districts in Bangladesh, and the obtained results are expected to be generalizable for the remaining territory of the country. The study findings carry public health importance in the establishment of potential surveillance for enhanced antimicrobial resistance and stewardship in human-veterinary interfaces.

Antimicrobials commonly used in animal husbandry and human health are more concerning in the context of zoonotic transmission of resistant bacteria. The discovery of *mcr* in chicken gut bacteria is of both national and global importance because of colistin's clinical applications^35^. Considerably large proportions of *E.coli* in the study were found to carry *mcr-1*, consistent with the multiple previous studies^7,36^. This study identified *mcr* at a higher frequency in *Salmonella* spp. than in *E. coli*, although colistin is not an antibiotic of choice for treating Salmonella infections. These salmonellae can be particular serovars that intrinsically remain resistant to colistin. Some earlier studies have identified group D serovars of Salmonella possessing O:9 somatic antigens confer natural colistin resistance^37,38^. However, this study did not confirm the serotyping of Salmonella interlinked to *mcr*-gene and colistin resistance. The natural reservoirs of colistin resistance may contribute to horizontal transmission of the resistant traits to surrounding susceptible isolates^39^. Furthermore, *mcr* presence was observed in *Proteus* spp. and *Klebsiella* spp. with a clear disparity between caged and cage-free chicken sources. The feeding types and nature of poultry chickens may contribute to differential gut flora isolates and their resistance attributes. Moreover, colistin use for treatment of poultry chickens may have contributed to the increased acquisition of *mcr* by the two types of bacteria. The other studies also showed that prior exposure was associated with high positive detection of *mcr* in the poultry isolates^11^.

The procedure used to collect data on antimicrobial usage history in this study had some inherent limitations. The previous colistin usage data provided by the chicken owners were self-reported and have not been evaluated in-depth. The drug-usage information collected from unskilled and less-educated workers may contain some information bias towards underreporting. A majority of the owners, managers, and workers in the PFs were untrained and uneducated enough to not understand the chemical types of antimicrobials. Sometimes they were unresponsive and unable to provide the generic names of the antimicrobial drugs that they used. Furthermore, neither the poultry farms nor the household chicken owners had an inventory and recording system for their applied therapeutic antimicrobials. Some chicken owners presented the commercial brand names of the antimicrobials used, or alternatively provided drug sachets or bottles directly to the field workers. The data enumerator entered the brand names of the antibiotics used as original data and generic names in parallel. Refusal and self-censoring were encountered occasionally. The factors together may have led to underreporting of and self-selection bias in the colistin usage data. There is a minute possibility of overreporting as our data enumerator had collected colistin treatment history only after checking validated evidence of the content. Therefore, the identified association between colistin exposure and *mcr* acquisition should definitely be valid. We anticipate an even stronger association if further cohort studies are designed with wide coverage of samples.

Unlike PFs chickens, the native chickens had less reported usage of colistin for treatment or growth promoter. Hypothetically, there should have considerably low phenotypic colistin resistance or *mcr* acquisition in the native chicken isolates. However, we detected a steady phenotypic resistance and *mcr*-carriage in the isolates of the native chicken group. There are several hypothetical explanations for these unanticipated outcomes. First, colistin usage history may be unreported by the home-based native chicken owners. Second, the native chickens may receive some feeds that were previously mixed with colistin as a growth-promoting agent, which was not known to the users. Similar evidence of extensive colistin use for growth promotion has been found in many countries^4^. Third, horizontal gene transfer (HGT) could explain the transmission of the *mcr* gene and associated phenotypic resistance from the poultry gut microbiota to the native chicken enteric flora^39^.

This study had a few more limitations. The investigation was conducted following a cross-sectional study-design, and no follow up was carried out due to resource limitations. This study analyzed five *mcr* gene variants such as *mcr-1* to *mcr-5*; some more recently reported variants, like, *mcr-6, mcr-7, mcr-8,* and *mcr-9* have not been investigated. Serological identification of *Salmonella* spp. has not been carried out. Salmonella serotyping, particularly against somatic O-antigen, could explain whether the identified bacteria were natural reservoirs of colistin resistance or carrying newly acquired resistance traits. Without the serotyping of Salmonella, the reporting of overall colistin resistance may become slightly overestimated. However, total Salmonellae identified in this study was around 10% only, which is unable to shift the whole study findings significantly. The small sample size was also a constraint to conducting fully powered statistical analyses. However, our results were generated from a resource-limited setting and maintained internal validity by repeating independent experiments where necessary. The findings have shed some light on colistin usage for animal husbandry, varieties of chicken-gut bacteria, their carriage of *mcr* genes, co-occurrences, and the genotypic-phenotypic association of colistin resistance. Further studies will be worthwhile to assess the external validity of our findings.

## Conclusions

The mobile colistin resistance genes circulate in bacteria isolated from both poultry chicken- and native chicken-droppings. *mcr-1* variant predominates in the Bangladeshi chicken gut bacteria over other variants, such as *mcr-2, mcr-3, mcr-4,* and *mcr-5*. The distribution of the *mcr* genes and phenotypic colistin resistance was moderately higher in poultry-chicken isolates. The prior use of colistin in chickens leads to increased acquisition of higher phenotypic resistance and *mcr* genes by gut-bacteria.

## Methods

### Study design and specimen collection

A cross-sectional study was conducted to examine *mcr-1* to *mcr-5* genes prevalence in bacterial isolates from chicken droppings. Poultry farms (PFs) were conveniently selected for chicken-faeces collection between July 2017 and June 2018. We selected 20 PFs from areas of the Dhaka, Manikgang, Gazipur, Tangail, and Mymensing districts, where major PFs are located in Bangladesh. Geographic information mapping software, ArcGIS version 10 for Windows, was used to draw a sampling spot-location map (Fig. 1). A structured questionnaire was administered to farm-owners to investigate the types of poultry chickens, their recent disease history, and records of antibiotics applied for treatment and prophylaxis. The study also made enquiries regarding the farm-owners’ levels of education and whether they had attained animal husbandry training. Native chickens are also a substantial and high demand meat source for the Bangladeshi population. People living in villages and sub-urban areas of the country raise native chickens on a non-commercial basis. The native chickens are maintained cage-free in the day time and are allowed to explore around the house to find natural foods. Sometimes commercial foods are provided. This study also examined the faeces of native chickens for bacterial isolates and for their carriage of the *mcr-1* to *mcr-5* genes and associated colistin susceptibility. The same structured questionnaire was administered to home-owners to seek information regarding the antibiotic exposure history of the native chickens. Data enumerators were trained beforehand on the use of the questionnaire by minimizing selection bias and interviewer bias.

### Bacterial isolation and identification

Chicken faecal samples were directly collected in specific specimen collection tubes following all safety precautions and aseptic techniques. For long distance transport, faeces samples were dipped into the Cary Blair transport medium (Oxoid, UK) before being transported to the laboratory. For bacterial isolation, approximately one gram of chicken-faeces was mixed in 400 μL of phosphate-buffered saline (PBS), and one loopful of diluted sample was streaked on MacConkey agar (Oxoid, UK) for growth of Gram-negative enteric bacilli. For the detection of *Salmonella* and *Shigella*, the diluted chick-droppings were enriched in Rappaport Vassiliadis soya broth (RVS Broth, Oxoid, UK) overnight and streaked separately on Salmonella-Shigella (SS) agar (Oxoid, UK) medium. After overnight incubation at 37 °C, distinct single colonies were picked and cultured again on tryptone soya agar (Lyophilchem, Italy) to prepare a pure-culture repository, which was stored at -80ºC. The purified bacterial colonies were identified by conventional biochemical procedures followed by a rapid biochemical-test kit (API 20E, BioMérieux, Durham, NC). Bacterial identification was validated further by 16S rDNA analyses.

### Antibiogram and minimum inhibitory concentration (MIC) assessment

The isolates' phenotypic antimicrobial susceptibilities were tested by the agar dilution method on Mueller–Hinton agar (MHA, Oxoid, Basingstoke, UK). The method further determined the lowest concentration of colistin (minimal inhibitory concentration, MIC) capable of inhibiting the visible growth of isolates^40,41^. Agar dilution was performed by incorporating different concentrations of colistin-sulfate powder (Santa Cruz Biotechnology Inc, TX) into an MHA medium from 1.0 to 128.0 μg/mL in a two-fold dilution series. Approximate twenty-milliliter volumes of MHA were used in 90-mm Petri dishes for agar dilution MICs. Each test inoculum was prepared by inoculating one pure culture colony into Mueller–Hinton broth and allowed incubation for three-hour at 37 °C that generates a density of inoculum about 10^4^ colony-forming units (CFU) per spot on the MHA. A 0.5 McFarland standard was used to visually compare a density equivalent to approximately 10^8^ CFU/mL. A micropipette was be used to inoculate plates and allowed the inoculum to dry on spots at room temperature. The plates were then inverted for incubation at 37 °C in air for 18–20 h. Agar dilution MICs were performed in duplicates. Several earlier studies conducted the epsilometer test (*E-test*) parallel to the agar dilution method to validate colistin MIC determination^42,43^. *E-test* was performed using a commercial strip containing a predefined gradient of colistin concentrations (Liofilchem Inc, Italy) to compare MIC with the agar dilution method^44^. *Escherichia coli* ATCC25922 was used as the susceptible-control reference strain for each batch of MIC tests. Also, a control plate without colistin-sulfate was tested for the growth of both test and control strains. The clinical breakpoints for colistin resistance were interpreted according to the European Committee on Antimicrobial Susceptibility Testing (EUCAST) guidelines when the MIC value was > 2 μg/mL^45^.

### Detection of the colistin resistance *mcr* gene variants

Multiplex polymerase chain reaction ((PCR) was conducted to detect the *mcr1 to mcr-5* genes in the isolates. The primer pair, CLR-F, (5′ CGGTCAGTCCGTTTGTTC 3′) and CLR-R, (5′ CTTGGTCGGTCTGTAGGG 3′), was used to yield a 309 bp DNA band for *mcr-1*, as described elsewhere^7^. The other four primer pairs to detect *mcr-2*, *mcr-3*, *mcr-4*, and *mcr-5* gene amplicons were obtained from another original study^20^. In brief, the modified protocol was as follows: prepared bacterial DNA (2.0 μL) was added to a 2× PCR premixture, (15 μL, GeneON, Germany) and five pmol of each primer (1 μL), and deionized water was added to obtain a final volume of 30 μL. Reactions underwent an initial denaturation at 94 °C for 15 min followed by 25 cycles of amplification (Applied Biosystems 2720 Thermal Cycler, Singapore), consisting of denaturation for 30 s at 94 °C, annealing for 90 s at 55 °C, and extension for 1 min at 72 °C, and a final 10 min elongation at 72 °C. Expected amplicons for *mcr-1* (309 bp), *mcr-2* (715 bp), *mcr-3* (929 bp), *mcr-4* (1116 bp), and *mcr-5* (1644 bp) were visualized under UV light after 1.2% agarose gel electrophoresis followed by staining with ethidium bromide. The obtained results were validated by separate singleplex PCR analyses of the *mcr*-genes.

### Sequencing of the *mcr-1* gene

Three randomly amplified *mcr-1* genes obtained by PCR were sequenced and blasted against the NCBI nucleotide database (https://blast.ncbi.nlm.nih.gov) to confirm their identity with other reported *mcr-1* genes. Sequence alignments were performed using the BioEdit program (version 7.2) for Windows. The revealed *mcr-1* sequences were submitted to GenBank, and the accession numbers obtained were MK615113, MK615114, and MK615116.

### Statistical analysis

A validated Bengali version of the questionnaire was used for data collection. Data collection supervisors rechecked completed data collection forms. In addition, 10% of the laboratory analyses were randomly selected twice by different researchers to assess inter-researcher biases. Verified data were entered and subsequently analysed using the IBM SPSS Statistics data editor (version 21). Missing data were excluded from the bivariate analysis. Descriptive and inferential statistical procedures were used to ascertain the types of chicken gut bacteria and their carriage of the *mcr* genes. Pearson's chi-square test was used to test the significance of association between categorical data, and Yate's correction for continuity was applied where appropriate. A two-tailed p-value small than 0.05 was considered to measure statistical significance.

### Ethics statement

This study was approved by the Ethics and Research Review Committee of the Jahangirnagar University Faculty of Biological Sciences [No. BBEC, JU/M 2017 12(4), dated December 27, 2017]. All methods were performed in accordance with the relevant guidelines and regulations. Informed consent was obtained from the respective farm owners or managers for collecting poultry droppings and information from their farms. Farm identities and information were kept strictly anonymous to protect their commercial, personal, and private information. The respective sample identification code was assigned duly for each sample collected.

### Conference presentation

ASM Biothreats on “Prophylactic Uses of Antimicrobials in Bangladeshi Poultry Drive Microbial Resistance (129)”, Baltimore, Maryland, February 12–14, 2018.

## Supplementary information

Supplementary information.

## Data Availability

A dataset was generated and analysed during the study that stored at the databank repository of One Health Laboratory, Department of Microbiology, Jahangirnagar University. Data can be shared upon request from the corresponding author following universal data-sharing. rules.
