# Supplementary material for: High abundance of the colistin resistance gene mcr-1 in chicken gut-bacteria in Bangladesh
Source: Sci Rep. 2020 Oct 14;10:17292. doi: 10.1038/s41598-020-74402-4 (PMC7560609; doi:10.1038/s41598-020-74402-4)
Supplement: Supplementary file 1 — Supplementary information. [file 41598_2020_74402_MOESM1_ESM.pdf]

High Abundance of the Colistin Resistance Gene *mcr-1* in Chicken Gut-Bacteria in Bangladesh

Running title: Antimicrobial resistance via zoonosis

Salequl Islam[1,*,∆], Umme Laila Urmi[1,∆], Masud Rana[1] , Fahmida Sultana[1], , Nusrat Jahan[1], Billal Hossain[1], Samiul Iqbal[2], Md. Moyazzem Hossain[3] , Abu Syed Md. Mosaddek[4] and Shamsun Nahar[1,*].

[1] Department of Microbiology, Jahangirnagar University, Savar, Dhaka-1342, Bangladesh.

[2] Department of Oral Maxillofacial Surgery, Faculty of Dentistry, BSMMU, Dhaka-1210, Bangladesh.

[3] Department of Statistics, Jahangirnagar University, Savar, Dhaka-1342, Bangladesh.

[4] Department of Pharmacology, Uttara Adhunik Medical College, Uttara, Dhaka-1230, Bangladesh.

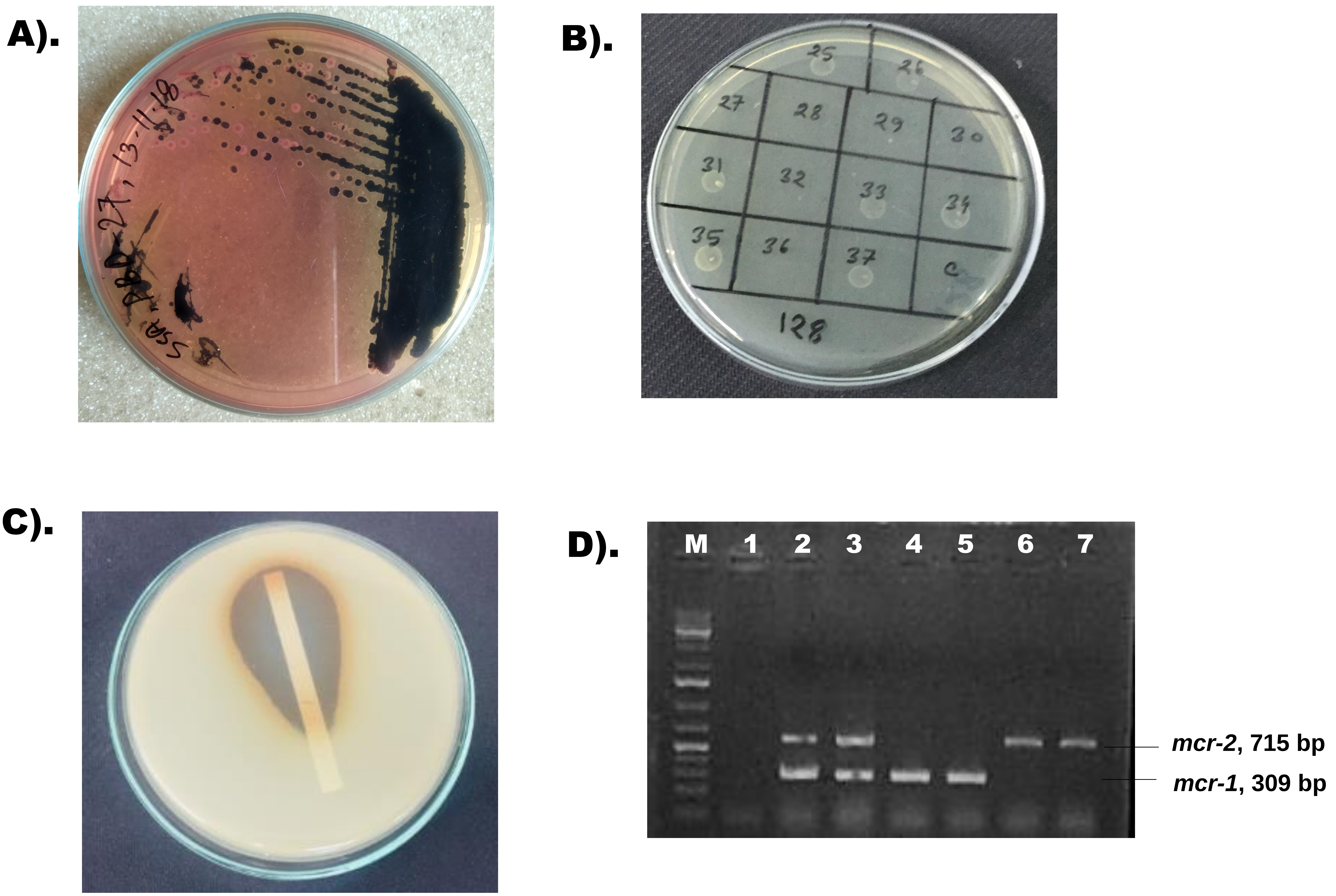

Supplementary Figure 1. Bacterial Isolation, Antibiotic Susceptibility Testing, and *mcr* Detection by Polymerase Chain Reaction (PCR).

A) Pre-enriched poultry droppings were inoculated onto selective Salmonella-Shigella (SS) agar medium. Visible bacterial growth was observed after overnight incubation at 37°C. B). For the susceptibility testing by agar dilution method, the colistin sulphate powder was dissolved in sterile distilled water and added to molten MH agar to 2-fold dilutions to make final concentration ranging from 1 µg/mL to 128 µg/mL. One petri-dish MHA plate with a specific concentration of colistin sulphate was divided into 14 spots manually. One microliter of a bacterial inoculum calculated approximate 10^4 CFU of bacteria was dropped on each spot and incubated 18-20 hours at 37°C. Following incubation, visible bacterial growth on plates on >2 µg/mL colistin sulphate was considered phenotypic resistance as per the European Committee on Antimicrobial Susceptibility Testing (EUCAST) guidelines. Similarly, the method further determined the minimal inhibitory concentration (MIC) for a particular isolate as the lowest concentration of colistin capable of inhibiting visible bacterial growth. C) MIC measurement using the commercial *Etest* (Liofilchem Inc, Italy). D) Amplified PCR products of *mcr-1* and *mcr-2* genes were 309 bp and 715 bp, respectively. The products underwent electrophoresis through 1.2% agarose gel and visualized under UV light. Lane 1 was negative-control for *mcr* genes. Lane 2 and 3 showed the overlapping presence of *mcr-1* and *mcr-2* genes. Lane 4 and 5 showed positive amplification of *mcr-1* gene; lane 6 and 7 endorsed the *mcr-2* gene. Lane 'M' shows a 1000 base pair DNA maker (Gene ruler, Thermo Fisher Scientific, MA).

A). Alignment of the nucleotide sequences of the mcr-1 gene from different colistin-resistant bacteria.

```
            10        20        30        40        50        60        70        80        90
C1 CAGCCAAACCTATCCCATCGCGGACAATCTCGGCTTTGTGCTGACGATCGCTGTCGTGCTCTTTGGCGCGATGCTACTGATCACCACGCTGT
C2 ............................................................................................
C3 ............................................................................................
          100       110       120       130       140       150       160       170       180
C1 TATCATCGTATCGCTATGTGCTAAAGCCTGTGTTGATTTTGCTATTAATCATGGGCGCGGTGACCAGTTATTTTACTGACACTTATGGCA
C2 ..........................................................................................
C3 ..........................................................................................
          190       200       210       220       230
C1 CGGTCTATGATACGACCATGCTCCAAAATGCCCTACAGA-CCGACCAAGAAGA
C2 .......................................-...........--
C3 .......................................A..........---
```

B). Alignment of the amino acid sequences of the mcr-1 gene from different colistin-resistant bacteria.

```
C1 METLLITTLLSSYRYVLKPVLILLLIMETGAVTSYFTDTYGTVYDTTMETLQNALQTDQE
C2 ............................................................
C3 ........................................................NRPR
```

Supplementary Figure 2. Sequence Alignment of the *mcr-1* Gene Identified from Different Colistin-resistant Bacteria.

A) Dots indicate the nucleotide identity among three partial sequences of the *mcr-1* gene identified in this study. Different letter letters represent differences among the amplified PCR products. B) Alignment of amino acid sequences of the partial *mcr-1* gene was detected in the study. Dots indicate identity and letters represent substitutions among the sequenced *mcr-1* products.

Supplementary Table 1. Inter-species phenotypic colistin susceptibility and *mcr* carriage in poultry- and native-chicken isolates.

Organism tested	Origin of isolates	Phenotypic colistin susceptibility by agar dilution, frequency (%) [a]			Carriage of *mcr* gene, frequency (%) [a]		
		Sensitive	Resistant	*p* value [b]	Yes	No	*p* value [b]
Proteus spp	Poultry-chicken	8 (22.2)	28 (77.8)	0.506	15 (41.7)	21 (58.3)	0.055
	Native-chicken	5 (31.3)	11 (68.8)		2 (12.5)	14 (87.5)	
Klebsiella spp	Poultry-chicken	6 (30)	14 (70)	1.00	9 (45.0)	11 (55.0)	0.101
	Native-chicken	3 (30)	7 (70)		1 (10.0)	9 (90.0)	
E. coli	Poultry-chicken	12 (57.1)	9 (42.9)	0.718	5 (23.8)	16 (76.2)	0.704
	Native-chicken	9 (69.2)	4 (30.8)		4 (30.8)	9 (69.2)	
Salmonella spp	Poultry-chicken	4 (40)	6 (60)	0.588	4 (40.0)	6 (60.0)	1.00
	Native-chicken	1 (16.7)	5 (83.3)		3 (50.0)	3 (50.0)	
Shigella spp	Poultry-chicken	1 (33.3)	2 (66.7)	-	1 (33.3)	2 (66.7)	-
	Native-chicken	0	0		0	0	
Enterobacter spp	Poultry-chicken	4 (44.4)	5 (55.6)	0.301	2 (22.2)	7 (77.8)	0.506
	Native-chicken	4 (80)	1 (20)		0	5 (100)	

[a], row percentage.

[b], p-value was calculated using Chi-square statistic of: i) between phenotypic colistin susceptible (MICs ≤2 µg/mL) and resistance (MICs >2 µg/mL) isolates, ii) between *mcr*-1-positive and *mcr*-1-negative isolates.

Supplementary Table 2. Association of *mcr*-genes with phenotypic colistin resistance.

**Colistin resistance gene**		**Phenotypic colistin susceptibility by agar dilution, frequency (%)** [a]		
		**Sensitive**	**Resistant**	***P* value** [b]
mcr-1	Positive (43)	0 (0)	43 (100)	*0.000*
(Excluding *mcr-2*)	Negative (103)	57 (53.8)	46 (46.2)	
mcr-2	Positive (5)	0 (0)	5 (100)	*0.064*
(Excluding *mcr-1*)	Negative (103)	57 (39.6)	46 (60.4)	
mcr-1+mcr-2 [c]	Positive (46)	0 (0)	46 (100)	*0.000*
	Negative (103)	57 (55.3)	46 (44.7)	

[a], row percentage

[b], p-value was calculated using Chi-square statistic.

[c], two isolates carried both *mcr-1* and *mcr-2* alleles.
